# Wanted a Home for a Lady with Creeping Paralysis

**Published:** 1886-10-09

**Authors:** Mary H. Mitcham

**Affiliations:** Surrey


### The Editor's Letter-Box.

[Our Correspondents are reminded that prolixity is a great bar to publication, and that brevity of style and conciseness of statement greatly facilitate early publication.]

---

### WANTED A HOME FOR A LADY WITH CREEPING PARALYSIS.

I am anxious to find a Home for a lady of limited means, who is suffering hopelessly from creeping paralysis. She has been living for some time in a Home for invalid ladies, but she now requires more nursing and care than can be given in an ordinary invalid Home, so I write to inquire if you can recommend a Home which receives patients of that kind, and should feel greatly obliged if you would kindly send me particulars, terms, etc.

Mary H.

Mitcham, Surrey, Oct. 2nd, 1886.

*** Can any reader suggest such a Home?—Ed. T. H.

